# A New Look at Familial Risk of Inflammatory Bowel Disease in the Ashkenazi Jewish Population

**DOI:** 10.1007/s10620-018-5219-9

**Published:** 2018-09-03

**Authors:** Elena R. Schiff, Matthew Frampton, Francesca Semplici, Stuart L. Bloom, Sara A. McCartney, Roser Vega, Laurence B. Lovat, Eleanor Wood, Ailsa L. Hart, Daniel Crespi, Mark A. Furman, Steven Mann, Charles D. Murray, Anthony W. Segal, Adam P. Levine

**Affiliations:** 1 Centre for Molecular Medicine, Division of Medicine, University College London, London, UK; 2 Department of Gastroenterology, University College London Hospital, London, UK; 3 Research Department of Tissue and Energy, Division of Surgery and Interventional Science, University College London, London, UK; 4 Gastroenterology Department, Homerton University Hospital, London, UK; 5 Gastroenterology Department, St Mark’s Hospital, London, UK; 6 Centre for Paediatric Gastroenterology, Royal Free Hospital, London, UK; 7 Gastroenterology Department, Barnet General Hospital, London, UK; 8 Centre for Gastroenterology, Royal Free Hospital, London, UK

**Keywords:** Epidemiology, Inflammatory bowel disease, Familial risk, Ashkenazi Jewish

## Abstract

**Background and Aims:**

The inflammatory bowel diseases (IBD) are particularly common among the Ashkenazi Jewish (AJ) population. Population-specific estimates of familial risk are important for counseling; however, relatively small cohorts of AJ IBD patients have been analyzed for familial risk to date. This study aimed to recruit a new cohort of AJ IBD patients, mainly from the UK, to determine the familial occurrence of disease.

**Methods:**

A total of 864 AJ IBD patients were recruited through advertisements, hospital clinics, and primary care. Participants were interviewed about their Jewish ancestry, disease phenotype, age of diagnosis, and family history of disease. Case notes were reviewed.

**Results:**

The 864 probands comprised 506 sporadic and 358 familial cases, the latter with a total of 625 affected relatives. Of the UK cases, 40% had a positive family history with 25% having at least one affected first-degree relative. These percentages were lower among those recruited through hospital clinics and primary care (33% for all relatives and 22% among first-degree relatives). Examining all probands, the relative risk of IBD for offspring, siblings, and parents was 10.5, 7.4, and 4, respectively. Age of diagnosis was significantly lower in familial versus sporadic patients with Crohn’s disease.

**Conclusions:**

This study reports familial risk estimates for a significant proportion of the AJ IBD population in the UK. The high rate of a positive family history in this cohort may reflect the greater genetic burden for IBD among AJs. These data are of value in predicting the likelihood of future recurrence of IBD in AJ families.

## Introduction

Crohn’s disease (CD) and ulcerative colitis (UC) are the two major forms of inflammatory bowel disease (IBD), a heterogeneous group of chronic and debilitating disorders characterized by inflammation of the gastrointestinal tract [1, 2]. IBD is a global disease, the prevalence and incidence of which have significantly increased since the 1950s, particularly over the last few decades in developed countries [3]. Recent estimates suggest that the prevalence of IBD is over 0.3% in North America, Australia, and many countries in Western Europe. IBD is also emerging in newly industrialized countries as they adopt a Westernized diet and lifestyle [4].

The etiology of IBD is thought to involve an aberrant immune response to commensal microflora in genetically susceptible individuals exposed to environmental risk factors [5, 6]. Epidemiological studies have consistently shown an increased prevalence of IBD among first-degree relatives (FDRs) of patients with CD and UC; this familial clustering, in addition to the increased incidence of disease among monozygotic (MZ) twins, provided the initial evidence of a genetic predisposition to IBD [7, 8]. Concordance in MZ twins is > 30% for CD and 16% for UC, while only 4% for both in dizygotic twins [9, 10]. A positive family history is the strongest risk factor for developing IBD; FDRs of an IBD patient have a 10–15 times greater risk of the disease compared with the general population [11, 12]. If both parents have IBD, the lifetime risk to their offspring is thought to be over 30% [13, 14].

The Jewish population comprises three main groups: Ashkenazim, Sephardim, and Mizrahim. The Ashkenazi Jewish (AJ) population is a founder population that comprises the majority of contemporary world Jewry. From the twelfth century, AJs lived in Central and Eastern Europe, moving primarily to the USA, Western Europe, and Israel in the late nineteenth and early to mid-twentieth centuries [15]. The AJ population was subjected to repeated bottlenecks and has maintained its genetic isolation through endogamy, practiced for religious and cultural reasons. This genetic isolation is evidenced by the high prevalence of founder mutations [16] corresponding to over 100 autosomal recessive disorders [17] as well as a high frequency of risk variants for common diseases such as breast and ovarian cancer, Parkinson’s disease, and IBD [18].

IBD is particularly common among the AJ population as demonstrated in epidemiological studies in disparate geographic locations since the 1960s [19–26]. The latest published data suggest an approximately fourfold increased risk of IBD among AJs [27]. There are no recent published epidemiological studies assessing the prevalence of IBD in the contemporary AJ population. Using an IBD prevalence of 0.3% [4], a fourfold increase among AJs yields an estimated prevalence of 1.2%. Previous studies reporting familial empirical risk estimates for IBD in the AJ population have been undertaken over 25 years ago using modest cohort sizes [26, 28].

The AJ population in the UK is thought to represent about 95% of the total UK Jewish population, which an analysis of the 2011 national census enumerated as 271,259 individuals [29]. The contemporary UK Jewish population is estimated to display a relatively high degree of endogamy at 74% [30]. Using an AJ IBD prevalence of 1.2%, there are an estimated 3100 AJ IBD cases in the UK.

The aim of this study was to recruit a new cohort of AJ IBD patients and their families, primarily from the UK, for epidemiological and genetic investigations [31]. Using 864 AJ IBD-affected individuals, up-to-date estimates of the pattern of familial disease and the risk to relatives are provided. Such data are relevant for genetic counseling.

## Methods

### Ethics

Ethical and research governance approval was provided by The National Research Ethics Service London Surrey Borders Committee (10/H0906/115) and the University College London Research Ethics Committee (6054/001). Informed consent was obtained from all participants.

### Recruitment

Individuals with IBD were recruited through advertisements in Jewish press, predominantly in the UK but also in Israel and the USA, and via referrals from specialist IBD clinics and primary care (UK only). Recruitment was undertaken from 2013 to 2017. Participants were interviewed by telephone to ascertain their Jewish ancestry, IBD phenotype, age of diagnosis, and family history of IBD in both FDRs and distant relatives. Only individuals of self-reported AJ ancestry were included; those with Sephardi, Mizrahi, or mixed ancestry were excluded. IBD cases were considered to have “sporadic” IBD if there were no known relatives with the disease. They were considered to have “familial” disease if a first, second, or more distant relative was reported to have a diagnosis of CD or UC. The diagnosis of IBD in probands (index cases) and their relatives was evaluated by asking them detailed questions about their diagnosis, clinical course, and treatment. Through the probands, the majority (66%) of their affected relatives were subsequently recruited from the UK and multiple countries worldwide including Australia, Belgium, Canada, France, Gibraltar, Israel, Netherlands, Norway, Portugal, South Africa, Switzerland, and the USA. Where possible a written confirmation of each affected individual’s diagnosis was obtained from his or her doctor. Sporadic and familial IBD cases were compared for IBD phenotype and age of diagnosis. Familial risk estimates were calculated. The ages of diagnosis in parent–offspring pairs was examined. Saliva samples were collected for genetic investigations.

### Statistics

2 × 2 comparisons were made using Fisher’s exact test (two-tailed). Multi-group comparisons for continuously distributed data were made using Tukey’s honest significant difference test. Single-group comparisons for continuously distributed data were made using Wilcoxon signed-rank test. Data analysis was performed in R.

## Results

### Participants

A total of 864 AJ index cases with IBD were recruited. Of these, 506 individuals had no family history of IBD (sporadic) and 358 reported one or more affected relative(s) (familial). In the families, there were a total of 625 additional affected relatives (301 FDRs and 324 more distant relatives); information was directly obtained from 411 of these. Of the total 1489 AJ IBD individuals in the cohort, 1261 (85%) were currently resident in the UK. Assuming a 1.2% prevalence of IBD in AJs, the cohort represents over 40% of the total predicted UK AJ IBD population.

### Distribution of Disease and Family History

The percentage of probands with a positive family history of IBD in any relative was 41% (Table 1). The number of probands with a FDR with IBD was 231 (27%). The percentage of probands in the UK with a positive family history in any relative was significantly greater when the proband was recruited through advertising (44%) than through hospital clinics and primary care (33%) (Fisher’s exact test *p* = 0.004) but not in FDRs only (*p* = 0.13). The percentage of probands with a positive family history in any relative and among FDRs only was significantly greater among probands recruited internationally (*p* = 0.002 and *p* = 0.003, respectively).

**Table 1:** Family history of IBD in all relatives and in first-degree relatives (FDRs) only of IBD probands, according to the method of recruitment

Recruitment method	IBD probands	Positive family history	
		All	FDRs only
Advertisements (UK)	529	231 (44%)	144 (27%)
Hospitals, primary care (UK)	276	91 (33%)	61 (22%)
Total (UK)	805	322 (40%)	205 (25%)
International	59	36 (61%)	26 (44%)
Total (overall)	864	358 (41%)	231 (27%)

Table 2 shows the distribution of all 1489 affected individuals by type of IBD in familial and sporadic cases; a greater number of affected individuals had CD than UC in both the familial and sporadic groups. Of the 864 probands, 528 (61%) had CD and 319 (37%) had UC. The remaining 17 (2%) had either a clinically indeterminate phenotype (IBD unclassified) or their subtype was unknown.

**Table 2:** Distribution of familial and sporadic patients by inflammatory bowel disease subtype in probands and the entire cohort

	Probands only (%)	All (%)
All IBD		
CD	528 (61%)	885 (59%)
UC	319 (37%)	532 (36%)
IBDU	17 (2%)	72 (4%)
Total	864	1489
Familial IBD		
CD	225 (63%)	582 (59%)
UC	130 (36%)	343 (35%)
IBDU	3 (1%)	58 (6%)
Total	358	983
Sporadic IBD		
CD	303 (60%)	
UC	189 (37%)	
IBDU	14 (3%)	
Total	506	

*CD* Crohn’s disease, *UC* ulcerative colitis, *IBDU* inflammatory bowel disease unclassified/unknown

A greater proportion of CD probands had an affected FDR (27%) than of UC probands (20%), *p* = 0.03. There was no significant difference in any positive family history among those with CD (43%) versus UC (41%), *p* = 0.62.

While affected relatives of a proband may develop either form of IBD, the greatest risk to a relative was for the concordant type, particularly for a CD proband (examining FDRs: 83% for CD versus 62% for UC, *p* = 0.0007). Table 3 shows the distribution of concordant and discordant FDRs of these probands.

**Table 3:** Distribution of all concordant and discordant affected FDRs of 209 CD and UC probands

Number of familial probands with ≥ 1 affected FDR by phenotype		Total number of affected FDRs by phenotype		
		CD	UC	Total
CD	144	*161 (83%)*	34 (17%)	195
UC	65	29 (38%)	*48 (62%)*	77
Total	209	190	82	272

Concordant phenotypes are indicated by italics

**Fig. 1:**
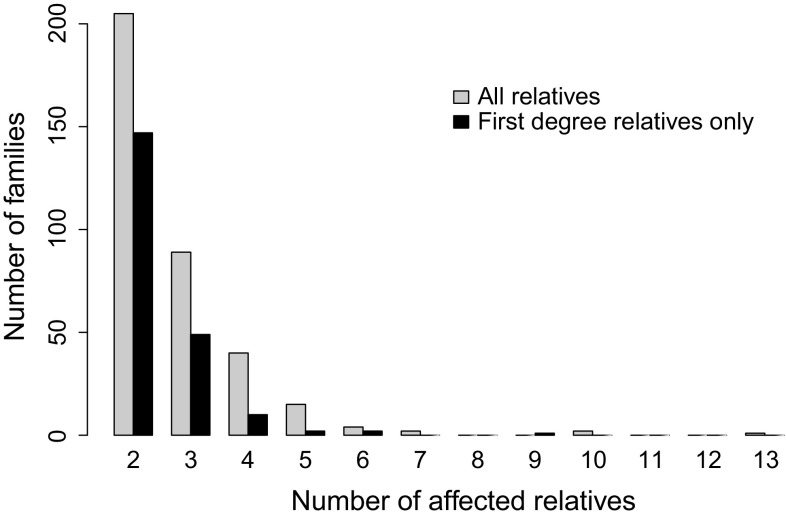
Distribution of the number of ascertained families by number of affected relatives (all and first-degree relatives only separately)

The distribution of the total number of ascertained familial affected individuals in each of the 358 multiplex families is shown in Fig. 1, separately for all affected relatives and for affected FDRs only. There were 24 families with five or more affected individuals. There were 146 CD or UC probands with affected relatives none of whom were FDRs. In at least 10 families, the disease was present in two or more unrelated branches of the family.

### IBD in Spouses

Eight probands had an affected spouse; five of the couples (62.5%) were concordant for IBD phenotype: four for CD and one for UC. The mean age of disease diagnosis was 23.9 in the first affected spouse and 24.8 in the second affected spouse. Only one of the 25 offspring of these eight couples was affected (diagnosed at 15 years of age); however, 18 of the remaining 24 offspring were under 20 years old at the time of ascertainment, and some of them may yet manifest IBD.

### Relative Risks in FDRs

Details of the number and affected status of all FDRs were provided by the majority of the 864 probands. In order to prevent an overestimation of the relative risk due to a potential biased inclusion of affected versus unaffected relatives, probands were excluded when the number and phenotype of all FDRs were incomplete. For two probands, there was no information regarding the parents. The frequency with which probands were excluded due to incomplete FDR information was greater in sporadic versus familial cases for both offspring and siblings (30.8% vs. 11.2% and 36.2% vs. 9.2%, respectively). Six hundred and sixty-eight probands had 738 offspring, of whom 93 (13%) were affected. Using the estimated prevalence of IBD in the AJ population of 1.2% (as above), the relative risk for offspring was 10.5. The relative risks for sibling and parents were 7.4 and 4, respectively. The likelihood of having a child with IBD was slightly greater if the proband had CD than if they had UC (14% vs. 9% for offspring), although not quite reaching statistical significance (*p* = 0.06). Table 4 summarizes these data, showing the percentage of offspring, siblings, and parents affected and the respective relative risks, for all IBD, for CD, and for UC probands. Relative risks or percentages of affected FDRs from other studies are provided for comparison. Examining only familial cases for IBD, the relative risks for offspring, siblings, and parents were 17.0, 12.9, and 9.7, respectively (data not shown).

**Table 4:** Calculation of relative risk of IBD in first-degree relatives (FDRs) of patients with IBD, CD and UC

	Relative type		
	Offspring	Siblings	Parents
All probands (*n* = 864; 506 sporadic, 358 familial)			
Total affected and unaffected, *t*	738 (668)	1407 (648)	1724 (862)
Number affected, *m*	93 (318)	125 (325)	83 (356)
Unaffected FDRs of sporadic probands	281 (350)	600 (323)	1012 (506)
Unaffected FDRs of familial probands	364 (318)	682 (325)	629 (356)
Percentage affected in all families, *f *= *m*/*t*	13%	9%	5%
Relative risk in all families, *r *= *f*/*p*	10.5	7.4	4
All CD probands (*n* = 528; 303 sporadic, 225 familial)			
Total affected and unaffected, *t*	478 (400)	962 (390)	1054 (527)
Number affected, *m*	69 (196)	90 (204)	51 (224)
Percentage affected in all families, *f *= *m*/*t*	14%	9%	5%
Relative risk in all families, *r *= *f*/*p*	12	7.8	4
All UC probands (*n* = 319; 189 sporadic, 130 familial)			
Total affected and unaffected, *t*	246 (257)	426 (248)	636 (318)
Number affected, *m*	23 (119)	34 (118)	31 (129)
Percentage affected in all families, *f *= *m*/*t*	9%	8%	5%
Relative risk in all families, *r *= *f*/*p*	7.8	6.7	4.1
Percentages of affected FDRs in other AJ studies Roth et al. [28]			
Uncorrected prevalence, *n* = 154	3%	5%	3%
Age-corrected prevalence, *n* = 154	9%	9%	4%
Yang et al. [26]			
Uncorrected prevalence in CD, *n* = 134	2%	8%	3%
Age-corrected prevalence in CD, *n* = 134	7%	17%	4%
Uncorrected prevalence in UC, *n* = 157	2%	2%	3%
Age-corrected prevalence in UC, *n* = 157	7%	5%	4%
Satsangi et al. [42]			
Relative risk of IBD, *n* = 433	24.7	12.5	4.4

The number of probands from whom FDR information was available is in brackets. *p*: assumed prevalence of IBD in AJ population, 1.2%

### Age of Diagnosis of IBD in Cases

**Fig. 2:**
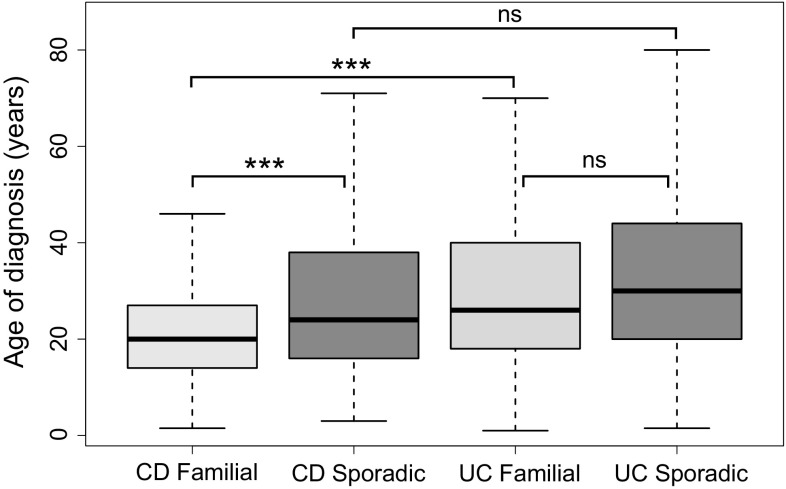
Box and whisker plots showing the distribution of age of diagnosis in Crohn’s disease (CD) and ulcerative colitis (UC) in familial and sporadic cases separately. *** *p *< 2 × 10^−5^, *ns* nonsignificant

The age of diagnosis differed significantly between CD and UC and familial and sporadic cases, as shown in Fig. 2. The data demonstrated a positive skew. The lowest age of diagnosis was observed in CD familial cases (median 20 years), and the greatest age of diagnosis was observed in UC sporadic cases (median 28.5 years). CD familial cases were statistically significantly younger at diagnosis than CD sporadic cases (*p* = 1.4 × 10^−5^) and UC familial cases (*p* = 2.5 × 10^−6^). The age at diagnosis of sporadic CD and UC cases, and UC familial and sporadic cases was not statistically different.

### Parent and Offspring Ages of Diagnosis

Of the 124 parent–offspring duos from whom ages of diagnosis were available, 79% of the children were diagnosed at a younger age than the affected parent, with a median age difference of 13 years (*p* = 3 × 10^−14^). This reduced to eight years but remained statistically significant (*p* = 1 × 10^−6^) when only parents diagnosed aged ≤ 40 years at the time of analysis were considered, in order to prevent an inadequate follow-up time bias of the offspring compared to the parents [32, 33].

## Discussion

This study reports updated familial risk estimates for the AJ IBD population utilizing a large newly recruited cohort, predominantly from the UK. Assuming a 1.2% prevalence of IBD in AJs, the 1261 AJ IBD individuals from the UK in this cohort represent a significant proportion (over 40%) of the estimated total UK AJ IBD population. The resulting risk estimates may be of benefit for genetic counseling. We report a 40% familial incidence of IBD in the UK with 25.5% of individuals with IBD having at least one first-degree relative with IBD. This is somewhat high in comparison with previous studies in non-AJ populations and is partly driven by the high number of familial cases who responded to our advertisements. Limiting the familial incidence estimates to cases recruited through hospitals and clinics (unbiased to family history status), we report a 33% familial incidence of IBD with 22.1% of individuals having at least one affected FDR. This matches well with results from a large recent study in the USA of 2136 patients with IBD (of variable ethnicity) which reported 32% familial incidence of IBD with 17% having at least one affected first-degree relative [34]. In a review of studies published up to 2002 [12], the occurrence of a positive family history of IBD varied from 5 to 16% in CD and 8 to 14% in UC. Large multigenerational studies from Sweden and Holland reported first-degree familial incidence of IBD in 6–12% [35, 36].

The rate of familial IBD observed in our cohort is greater than that of previous studies examining the AJ population (Table 5). However, it is important to note that there are challenges when comparing different studies given differing study designs and ascertainment methodologies. Defining familial IBD varies with some studies reporting family history in FDRs only and others including more distantly related relatives (e.g., second and third degree), as has been done in the present study. Some studies assign the risk of CD or UC [37–40], while others assign a risk to all types of IBD together [26, 28].

**Table 5:** Comparison between the present cohort and previous studies on the occurrence of familial IBD in AJs, showing occurrence of any affected relatives and affected first-degree relatives in probands with IBD

Study	Phenotype	Number of AJ probands	Any family history	FDR with IBD
Roth et al. [28]	IBD	154	23.4%	17.6%
Zlotogora et al. [37]	CD	157	NA	6.6%
Yang et al. [26]	IBD	291	24.3%	NA
Zhou et al. [38]	CD	481	22.8%	NA
Ben-Horin et al. [39]^a^	CD	181	NA	16%
Ben-Horin et al. [39]^a^	UC	168	NA	14%
This study—total	IBD	864	41%	25%
This study—UK	IBD	805	40%	26%
This study—UK	CD	484	41%	28%
This study—UK	UC	304	41%	22%
This study—UK Hospitals and primary care only	IBD	276	33%	22%

^a^The studies by Ben-Horin et al. examined the Israeli Jewish population; approximately 40% of the probands were Ashkenazi

Accurate empirical risk estimates are one of the most important tools for genetic counselors in supporting families and explaining complex diseases such as IBD [41] where the collective effect of multiple genetic loci has not been fully determined. The empirical risk estimates for siblings are more reliable compared to those for offspring or parents because probands and their siblings live temporally, removing generational bias [26]. Parents consistently have the lowest risk estimates, perhaps due to improved diagnosis and consistent with the increasing incidence of IBD in recent years. The relative risk for parents in our study was consistent with the literature [28, 42].

It has previously been observed that familial IBD cases tend to have an earlier age of onset compared to sporadic cases [34, 38, 43] although not all studies agree [44]. We observed this in our cohort for CD but not UC. Recent studies demonstrate that positive family history is also associated with complicated CD behavior and the need for IBD-related surgery [34, 45, 46], indicating the importance of establishing a family history for clinical and treatment prognostication. Information regarding disease severity was not collected in this study.

Our observation of a generational difference in the age of diagnosis in parent–offspring duos is consistent with previous studies in IBD [47–49]. Genetic anticipation as an explanation for this finding has been shown to be unlikely, and it is thought instead that ascertainment biases, especially inadequate follow-up time, are responsible [33, 50, 51].

Limitations of this study include potential biases and inconsistencies introduced in the recruitment process. The majority of affected individuals and their respective families were recruited through advertisements in the Jewish press; the second largest recruitment source was IBD clinics from five London hospitals in areas with a high Jewish population. As teaching hospitals, the latter are more likely to attract patients with severe disease, more common in familial cases [34]. Two potential issues with recruitment by advertisements are worth noting. Firstly, in contrast to the IBD clinics, participants responding to the advertisements self-reported their phenotypes. In the majority of cases (56%), the phenotype was independently verified by the patient’s clinician but this was not always possible. Self-reported IBD status has previously been found to be highly accurate [52, 53]. The other issue with recruitment by advertisement relates to the potential bias toward familial IBD. Since the aim of this study was also to identify multiplex families for genetic analysis, the advertisement particularly encouraged participation of such cases. This is evident in the data in which the percentage of probands with a family history was greater among those recruited by advertisement. It is also possible that there was more of an incentive to participate in a genetic study for familial cases.

Furthermore, we have also relied on probands self-reporting a positive or negative family history. The reliability of this has not been previously studied for IBD, although it has been demonstrated to be accurate for some cancers [54, 55]. Lack of contact with relatives may cause underreporting of IBD in a family, while ignorance of diagnosis may cause over-reporting.

The lifetime risk of IBD in the probands’ relatives has not been adjusted for age. Since many of the relatives are young, they may still develop IBD. The relative risks calculated may thus be underestimated, although the aforementioned biases may counteract this.

This study characterizes a newly recruited AJ IBD population and examines the distribution of family history in detail. The relatively high rate of a positive family history in this cohort may reflect the greater genetic burden for IBD among AJs [56, 57]. Such data and the relative risk estimates are of value in predicting the likelihood of future recurrence of IBD in a family.

## Abbreviations

AJ: Ashkenazi Jewish
FDR: First-degree relative
